# Unmet Needs in Patients With Heart Failure: The Importance of Palliative Care in a Heart Failure Clinic

**DOI:** 10.3389/fcvm.2022.866794

**Published:** 2022-05-30

**Authors:** Valentina Gonzalez-Jaramillo, Maud Maessen, Nora Luethi, Jelena Guyer, Lukas Hunziker, Steffen Eychmüller, Sofia C. Zambrano

**Affiliations:** ^1^University Center for Palliative Care, Inselspital, University Hospital Bern, University of Bern, Bern, Switzerland; ^2^Institute of Social and Preventive Medicine (ISPM), University of Bern, Bern, Switzerland; ^3^Department of Emergency Medicine, Inselspital, University Hospital Bern, University of Bern, Bern, Switzerland; ^4^Department of Pediatrics, Hospital of Biel, Biel, Switzerland; ^5^Department of Cardiology, Inselspital University Hospital Bern, Bern, Switzerland

**Keywords:** heart failure, palliative care, patient-centered care, qualitative study, multidisciplinary team, quality of care/care delivery

## Abstract

**Background:**

There are increasing calls to establish heart failure (HF) clinics due to their effectiveness in the interdisciplinary management of people living with HF. However, although a recommendation exists for palliative care (PC) providers to be part of the interdisciplinary team, few of the established HF clinics include them in their teams. Therefore, in this qualitative study, we aimed to understand the unmet PC needs of patients with HF attending an already established HF clinic.

**Methods:**

Secondary qualitative analysis of structured interviews undertaken within a larger study to validate the German version of the Needs Assessment Tool: Progressive Disease—Heart Failure (NAT: PD-HF). The NAT: PD-HF is a tool that aims to assess unmet needs in patients with HF. The interviews took place between January and March 2020 with patients from the ambulatory HF Clinic of a University Hospital in Switzerland. For this analysis, we transcribed and thematically analyzed the longest and most content-rich interviews until we reached data saturation at 31 participants. The interviews lasted 31 min on average (24–48 min).

**Results:**

Participants (*n* = 31) had a median age of 64 years (IQR 56–77), the majority had reduced ejection fraction, were men, and were classified as having a New York Heart Association functional class II. Participants were in general satisfied with the treatment and information received at the HF clinic. However, they reported several unmet needs. We therefore identified three ambivalences as main themes: (I) “feeling well-informed but missing essential discussions”, (II) “although feeling mostly satisfied with the care, remaining with unmet care needs”, and (III) “fearing a referral to palliative care but acknowledging its importance”.

**Conclusion:**

Although patients who are receiving multidisciplinary management in ambulatory HF clinics are generally satisfied with the care received, they remain with unmet needs. These unmet needs, such as the need for advance care planning or the need for timely and tactful end-of-life discussions, can be fulfilled by PC providers. Including personnel trained in PC as part of the multidisciplinary team could help to address patients' needs, thus improving the quality of care and the quality of life of people living with HF.

## Introduction

For several decades, evidence has shown that patients with heart failure (HF) not only have palliative care (PC) needs comparable to patients with cancer but often for a significantly longer period of time (1). However, in most countries, access to PC for patients with HF is still suboptimal and gaps between PC needs and PC delivery within this patient population are substantial (1, 2). In the 2019 European atlas of PC services (3), only eight European countries reported to have cardiology services that provided PC: the Czech Republic, Denmark, Ireland, the Netherlands, Portugal, Spain, Sweden, and the United Kingdom. The atlas also identified that collaboration between cardiology services and PC specialists occurs only occasionally (3).

Due to the complex needs of patients with HF, HF clinics, consisting of a multidisciplinary team that includes cardiologists, HF-trained nurses, internists, nutritionists, psychologists, physiotherapists, and social workers, are being established across the world to improve patient's HF management (4). According to evidence from observational studies and clinical trials, HF clinics are effective in reducing hospitalizations and all-cause mortality when compared to usual care (5–7). However, while these clinics are widely available in countries such as Norway and Italy, the current number of HF centers in other countries may not be sufficient to ensure a comprehensive evaluation according to current standards and recommendations (3).

Therefore, more HF clinics and multidisciplinary HF programs will need to be created in the future. Consequently, understanding the PC needs of patients seen in HF clinics, as well as the potential role for PC specialists or of PC-trained staff as part of these teams is crucial. Thus, we conducted this study to understand the unmet PC needs of patients with HF attending an already established HF clinic in a tertiary university teaching hospital in Switzerland.

## Materials and Methods

### Study Design

This is a secondary qualitative analysis of structured interviews undertaken within a larger study (8) to validate the German version of the Needs Assessment Tool: Progressive Disease—Heart Failure (NAT: PD-HF). After obtaining ethical approval (#2018-02175) for this analysis, we transcribed and thematically analyzed the longest interviews, as they were the most content-rich because participants engaged in deeper discussions with the interviewer. Before transcription, we heard all interviews and noticed that the shortest ones did not allow for a comprehensive understanding of participants' experience of symptoms and of their unmet needs and therefore decided that not all could be analyzed in the depth that thematic analysis requires. We then included interviews until we reached data saturation, that is, the point at which the inclusion of additional interviews would have not added substantial new information to the analysis and the resulting themes and subthemes (9). Of the 70 interviews undertaken for the validation study, we reached data saturation at 31 interviews. The interviews included in this study lasted 31 min in average (range: 24–48 min).

### Setting

The interviews took place between January and March 2020 with patients from the ambulatory HF clinic of the Inselspital, the University Hospital of Bern, Switzerland.

The HF clinic was created in the 1990s in the context of heart transplantation. Currently, it receives around 2,800 consultations per year and offers multidisciplinary care including cardiologists, HFnurses, cardiac rehabilitation, and cardiopsychology. The frequency of consultations depends on the stage and progression of the disease. Some patients may have consultations every month, every 3 months or even annually as in the case of transplant patients. Although the cardiopsychology service is open to all patients, a cardiopsychological assessment is routine and mandatory only before transplantation. As a means of information, brochures with different information related to HF are available for all patients. There are brochures with general recommendations on lifestyle, others explain what HF is, etc. There are no brochures with information on palliative care.

### The Needs Assessment Tool: Progressive Disease-Heart Failure

The NAT: PD-HF (10) is a tool that aims to assess unmet needs in patients with HF and their informal caregivers. The tool was created to be filled out by a health care professional and evaluates different types of needs, including physical, psychological, social, and spiritual needs. Additionally, the NAT: PD-HF assesses information needs including need for information about disease prognosis, treatment options, advance directives, health support services, financial or legal issues, and social or emotional issues (Supplementary Material 1). The tool consists of four parts: (1) priority referral for further assessment, (2) patient well-being, (3) ability of the informal caregiver or family to care for the patient, and (4) caregiver well-being. For each question, the health care professional undertaking the assessment together with the patient selects the level of concern: (1) none, (2) some/potential, (3) significant.

### Participants and Study Procedure

Patients were eligible to participate if they were adults (≥18 years old), able to communicate fluently in German and had a follow-up appointment at the HF clinic after having had at least one previous consultation at the clinic before. No stage or severity of HF was preselected as inclusion criteria, to ensure a complete and representative spectrum of patients at different stages of the disease. Patients who met the inclusion criteria were invited to participate via mail. For those who were interested, we arranged an appointment with a study member immediately before or after their scheduled consultation at the HF clinic. In the invitation package that was mailed to patients, we mentioned that the project aimed to evaluate the performance of a questionnaire to assess the needs of patients with HF and to improve the quality of care delivered to this population. We assessed patients' cognitive ability by asking them three questions about the study after explaining its purpose and the contents of the consent form and we excluded participants with poor comprehension capacity. Following participant consent, the interviews were conducted in Swiss German by JG, a 6th year female medical student. The interview addressed potential unmet needs of patients with HF and their family treated in an ambulant HF clinic. The interviews often focused on the care from the HF clinic but the experiences reported by the patients were not restricted to this care setting. All interviews covered the same questions and explored the areas in the same order as in the tool (refer to Supplementary Material 1 for a copy of the tool used to guide the interviews). In addition, when necessary, the interviewer went deeper into some of the issues and needs reported by the patients in order to select the level of concern, which may help explain the variation in the length of the interviews. For the qualitative analysis, a Swiss German linguist specialized in English language and literature, translated and fully transcribed all interviews to English from voice recordings.

### Data Analysis

We analyzed the interview transcripts via thematic analysis following Braun and Clarke (11), using NVivo software (12). VGJ, a female physician who was completing a PhD on PC in HF at the time of the study, performed all analyses with support from SCZ, a female psychologist with expertise in qualitative methods and PC research. VGJ and SCZ coded independently one of the interviews and compared all initial codes, to fine-tune the coding process. The coding comparison was made to ensure that VGJ was noticing all meaningful instances to be coded and to show the differences between semantic and latent coding, so that all subsequent interviews were coded in a similar way. At all times during the coding phase, SCZ and VGJ held discussions about how to code specific segments of data, when VGJ raised questions. Subsequently, and after familiarizing herself with all the interviews by reading and rereading the interview transcripts, VJG inductively assigned initial codes to all other interviews by staying close to the data and by keeping an audit trail with detailed notes about her assumptions and perceptions of initial thematic contents. The initial codes were either semantic or latent, meaning that at times portions of data were coded by explicitly describing what the participants stated (semantic coding), while the latent codes involved labeling the data with interpretations of underlying contents in participants' statements. Neither types of codes were given more priority than others in subsequent stages of analyses, though there were more semantic than latent codes across the dataset. In a second step, each of the codes was categorized under greater thematic categories, which were constantly defined and refined to fit the themes to the whole corpus of data. This process involved merging similar codes, renaming other codes, as well as establishing a hierarchy within the codes so as to promote some as themes and other as subthemes, always taking into account the different views expressed by participants across all interviews. Similarly, candidate themes and subthemes were then closely observed and reorganized to determine how well they represented the aspects discussed in all interviews, as well as cases which were less common across the dataset. In the last stage, VGJ and SCZ reviewed and agreed on the final themes via the same process of constant comparison and making final decisions about which themes allowed for the most comprehensive understanding of participants' accounts. At this stage, we also selected the most representative participant quotes to illustrate the themes and subthemes. We conducted, analyzed and report the study results according to the Consolidated Criteria for Reporting Qualitative Studies (COREQ) guidelines (13). In reporting the results, instead of presenting frequencies, we use terms such as “most” and “some,” as suggested by Braun and Clarke (12).

## Results

### Participants

The majority (*n* = 26) of the 31 participants were men. Participants had a median age of 64 years (IQR 56–77) and the majority had reduced ejection fraction and were classified as having a New York Heart Association functional class II. Twelve participants (39%) had an implantable cardioverter defibrillator, six (19%) had a ventricular assist device, and five (16%) were listed for a heart transplant (refer to Table 1). The distribution of clinical and sociodemographic characteristics were similar to those of the whole study population that participated in the validation study of the German version of the NAT: PD-HF). Even the proportion of women in our secondary analysis was equally low to the proportion of women in the original cohort (Supplementary Material 2). During the recruitment process, no patients were excluded due to poor comprehension capacity.

**Table 1:** Characteristics of the patients included in the study (*n* = 31).

**Characteristics**	***n* (%) or median (IQR)**
**Sex**	
Women	5 (16%)
Men	26 (84%)
**Age**	64 (56–77)
**LVEF (%)**	30 (20–50)
**LVEF category**	
HFrEF	18 (58%)
HFmrEF	8 (26%)
HFpEF	5 (16%)
**NYHA functional class**	
I	8 (26%)
II	14 (45%)
III	9 (29%)
**ICD**	12 (39%)
**VAD**	6 (19%)
**Heart transplant list**	5 (16%)
**COPD**	6 (19%)
**T2DM**	9 (29%)
**CAD**	13 (42%)
**CKD**	17 (55%)

*IQR, interquartile rate; LVEF, left ventricular ejection fraction; HFrEF, heart failure with reduced ejection fraction; HFmrEF, heart failure with mildly reduced ejection fraction; HFpEF, heart failure with preserved ejection fraction; NYHA, New York Heart Association; ICD, implantable cardioverter defibrillator; VAD, ventricular assist device; COPD, chronic obstructive pulmonary disease; T2DM, type 2 diabetes mellitus; CAD, coronary artery disease; CKD, chronic kidney disease*.

### Themes

Participants were in general satisfied with the treatment and information received at the ambulatory HF clinic, however, upon further questioning, they would often report several unmet needs. We therefore identified three ambivalences as main themes: (I) “feeling well-informed but missing essential discussions,” (II) “although feeling mostly satisfied with care, remaining with unmet care needs,” and (III) “fearing a referral to palliative care but acknowledging its importance.”

#### Feeling Well-Informed but Missing Essential Discussions

The majority of participants reported very often that they felt well-informed. Yet, as the assessment tool guiding the interviews covered different topics, several unaddressed issues surfaced during the interviews. Within this theme, we identified four subthemes: (a) *Feeling well-informed about their diagnosis, treatment options, medication and medical support*, (b) *Lacking information about advance directives, prognosis or about concurrent palliative care*, (c) *Being confronted with end-of-life discussions in an untimely and tactless manner*, and (d) *Preferring other communication channels*.

- *Feeling well-informed about their diagnosis, treatment options, medication and medical support:* The majority of patients considered that they had a good knowledge about their diagnosis of HF and the course of the disease. However, none of the patients elaborated on what they know about their disease or its course. When asked whether they would like to receive more information from the health care professionals on any of those aspects, none of them felt in need of more information and mentioned that if they needed it, they knew they could ask their cardiologist or said they could look for this information from other reliable sources. Participants also knew details about different treatment possibilities, mainly about medications or the option to have a cardiac device implanted. Some of the participants mentioned that although they did not know in detail how each of the medications worked specifically, they felt well-informed about the correct way of taking them.
  *Interviewer: Would you need more information about your heart disease, next steps or treatment options?*
  *Participant: Not really. I'm well-informed, I also know about the course of my heart disease. [...] The doctors here at (hospital) always told me you could still do this or that. And then you can still decide on your own whether you want it or not. (P26), male, 81 years old, NYHA III*.
- *Lacking information about advance directives, prognosis or about concurrent palliative care*: Despite believing they had a good knowledge of their disease, when it came to its prognosis, some patients mentioned they lacked prognostic information and felt that the physicians at the HF clinic often avoided this topic. Other participants felt that they needed to obtain more concrete information about how to fill an advance directive or about non-curative options. Others, more than needing information on how to fill out an advance directive, wanted pragmatic help completing it. When asked if they had ever been referred to PC, none of the participants had been referred to PC in the past, which also brought about their complete lack of understanding of PC concepts and about concurrent PC. We observed this unfamiliarity at three levels: some participants were unfamiliar with the term PC, others said they had never received it because they were ‘not at that stage' or because they still had curative therapeutic options available to them, and finally, because some thought that PC was only for patients with cancer and did not know it was available for patients with HF. Although some patients reported that “they were not at that stage”, they did not specify when they would consider themselves in need of palliative care.
  *(obtaining information about) the prognosis is difficult. I tried to ask today. The doctors avoid it of course, that's clear*. (P46, male, 79 years old, NYHA II).
- *Being confronted with end-of-life discussions in an untimely and tactless manner*: Some patients mentioned that when being hospitalized for an acute decompensation, they felt confronted when physicians discussed end-of-life issues with them in the intensive care unit or in another inpatient setting, perceiving these conversations as untimely and tactless. Participants did not seem in fear of the end-of-life discussions *per se* but felt that having them during an acute crisis was not appropriate. In many cases, these were the first instances when participants were confronted with end-of-life discussions while the physicians seemed to be in urgency to find out what to do or not to do should the patient be dying during their shift. One participant said that while he prefers direct conversations to having information withheld from him by health care staff or his family, he found the conversation while he was in the ICU to be crude and “*hated it*.” For another participant, this conversation came late at night during the hospitalization leaving her shocked and scared of falling asleep for fear of dying in her sleep. They did not elaborate on how they would like these discussions to go or when they would have preferred to have had such discussions.
  *So, I was up there in intensive care. And then they said very clearly: “what do you want us to do? It could turn out this or that way, how far should we go?” That was brutal, I thought, “hey, you could word that a bit differently”, I thought that was like a shock, that whole story*. (P12, men, 53 years old, NYHA II).
- *Preferring other communication channels:* When discussing different information needs, such as treatment options, health support services, or emotional support, many patients mentioned receiving leaflets or brochures covering these and many other topics. Although they received these brochures and felt that these contained relevant information, the majority of participants had strong preferences for having additional, more personalized and interactive communication channels with the treating team. They mentioned that having a pile of brochures was not of real help, and that they perceived brochures and leaflets as tactless and impersonal. In addition, some reported that certain information they encountered when reading the brochures at home caused them anxiety or confusion and that they often had to wait for answers from their cardiologist during a later in-person consultation, which could still have been months away.
  *I would not want to be overwhelmed with material, without me doing anything about it. [...] in the hospital back then, those 3 days, you have—everything went quite quickly, you got that brochure, that booklet and then a woman came into the room, pretty much gave the quick run-through: Like this, this, this, here's the material, have a nice day. There I would wish that the person that's doing that would take a bit more time. Maybe more of the interpersonal, too, not just following a set pattern. That way you can probably reach a patient better and then, yes. (P32, male, 52 years, NYHA I)*.

#### Although Feeling Mostly Satisfied With Care, Remaining With Unmet Care Needs

Several patients had a general feeling of being well-supported in the HF clinic and felt well-cared for by the HF team. However, some participants reported dissatisfaction with the care they received, including lack of support mainly in other hospital settings when they had a HF decompensation or at other crisis points. Within this theme, we identified four subthemes: (a) *Trusting the ambulatory heart failure team and having a general feeling of being well-supported*, (b) *Feeling alone and without support*, 3) *Feeling unheard when voicing specific symptoms*, and (c) *Lacking continuity of care*.

- *Trusting the ambulatory heart failure team and having a general feeling of being well supported*: We found that patients had a general feeling of being well-supported in the HF clinic and that they acknowledged the benefit they derived from the support of the different areas of expertise covered by the clinic and the staff members. For example, the support of the psychologist was seen as important not only for addressing non-physical symptoms, but also in their role as a mediator between the physician and the patient.
  *They (the doctors) were always transparent with me, they always showed me where I'm at and because of that I have never experienced anything negative but always positive. And it was always clear to me how it would go on, what we are doing as a next step, so from that side I've always had great support up to now. I can't complain at all, that's probably why I'm sitting here, because of this support. I've profited a lot. And I can only say positive things, I can only give praise. [...] I was always really happy with the work of (name) and Dr. (name). From both sides I always received all the information. Additionally, I get this psychological support. When something was unclear on the somatic side, I could also talk to the psychologist, then she was also a mediator between patient and doctors. And that always worked really well, that was really great work. (P43, female, 50 years old, NYHA II)*.
- *Feeling alone and without support*: At specific times during the illness trajectory, some participants felt that they needed more support and mentioned feeling alone. For example, two patients reported feeling lonely and hopeless after discharge. Another participant mentioned feeling a lack of support in the process with the ventricular assist device both before implantation due to lack of information and discussions, and after implantation, in the process of adapting to living with the device. For another participant there was a need for religious support while being hospitalized, and for another one, the need for emotional support after an intense suffocation event. Finally, another participant felt unsupported when, after an acute event, he was referred to a cardiac rehabilitation clinic even though he still felt very weak physically and mentally. Situations like these made the participants feel vulnerable and without a key contact person who could offer support during those times.
  *It was also—You had to wait for the pacemaker for 3 months, right? And then I always felt like those 3 months, they're waiting to see if you survive or not. [...]Yes, yes, it was about that because the device I have is quite expensive and then they wanted to see if “that dude will even make it” [laughs]. And “if he makes it, you can give him the pacemaker” right? And otherwise, the money is thrown out the window, kind of like that. Then I have to say, yes, that gets to you, that gets to you! Although, it does make sense, I have to say. Why should you install an expensive device if he doesn't make it anyways? But for the individual it's hard to take and you're left alone with that. You're just sent home, now [wait and] see. (P8, male, 61 years old, NYHA II)*.
- *Feeling unheard when voicing specific symptoms*: Some patients referred feeling unheard when voicing specific symptoms or needs, particularly when these were not caused by the disease itself, but were secondary to treatments for the disease. This was the case for example, when participants reported having back pain due to the weight of the ventricular assist device or when they found it cumbersome to carry out day-to-day activities with these devices. Other participants sought support for symptoms such as nausea, and often reported receiving no specific treatments for these. This often led them to feelings of disappointment or despair as they expected that these symptoms and concerns should have also been addressed in the HF clinic.
  *I of course have back pain. I always have to carry around these devices. No one is interested in that here. The (hospital name) doesn't give a [coughs] about this. Did you hear that? Little machine? That would be something that I thought would be important to have support with that—(P21, female, 57 years old, NYHA II)*.
- *Lacking continuity of care*: The consultations in the HF clinic were often performed by different physicians. For some of the patients it was uncomfortable to have a new physician at each follow-up consultation because they felt there was no time to develop a relationship with the physician.
  *What takes some getting used to is that there's always someone new, that there is not one (person)- Here, again, it was someone different who did the consultation hour. [...] It's pretty interesting, you get to meet different people all the time, but you don't actually get to know them, it's so quick. (P32, male, 52 years old, NYHA I)*.

#### Fearing a Referral to Palliative Care but Acknowledging Its Importance

We identified that patients feared PC referrals because they associated these referrals with death or with “when there was nothing else to be done.” However, while the term could generate some discomfort, participants were still able to understand the value of such an approach and seemed open to receiving PC later, especially after a brief explanation of the interviewer about what concurrent and early PC is. We identified three subthemes under this theme: (1) *associating palliative care with end of life and loss of hope*, (2) *acknowledging the importance of palliative care*, and (3) *explaining concurrent palliative care has the potential for positive impact*.

- *Associating palliative care with end of life and loss of hope*: The majority of the patients associated PC with care at the end of life or thought of it as a last resort for when there were no more curative therapies to try. For some of the participants, this negative perception of PC stemmed from previous experiences with family members or acquaintances. For example, one patient associated PC with when his father with cancer was in the terminal phase. Another patient recalled hearing the term when during a hospitalization, the doctors told another patient he shared the room with, that they could no longer offer him curative therapies and therefore would move him to the PC ward. Many were thus misinformed about PC and therefore a referral to PC was associated with dying and death.
  *I've heard of it, it's just the last thing, when there's nothing, no rescue, so to say. (P45, male, 71 years old, NYHA II)*.
- *Acknowledging the importance of palliative care*: We found that several participants despite giving a negative connotation to PC, also associated it with symptom relief, particularly for the treatment of pain or for maintaining quality of life. One participant said that she was aware of the benefits of PC but because of the connotation it has, she felt that if referred to PC, it would have a negative psychological impact for her.
  *And it's certainly a good thing and when there's nothing else, it's certainly better than doing all kinds of stuff, with medication for and against it and the third one so that it can be tolerated and so on. And at some point you might have to say “no, that was it.” (P50, male, 81 years old, NYHA III)*.
- *Explaining concurrent palliative care has the potential for positive impact:* After a brief explanation of the definition of concurrent and early PC, almost all participants thought the concept was interesting and expressed a desire to know more about it, as they could understand that it was not about dying, but about living with the disease. One of the participants associated it with psycho-cardiology, and another one thought that the option should be supported more within HF. Only one participant was the exception to this, as despite understanding what concurrent PC is, he reported not being interested in receiving this type of care.
  *I can imagine, the way you described it now, that this could make sense. Or that this would have potential to build on, as a form of support. Not in view of dying but more for living. (P56, male, 77 years old, NYHA II)*.

## Discussion

### Key Findings

Overall, participants were satisfied with the information and care received at the ambulatory HF clinic. However, even though the majority of the patients in our study were not at an advanced stage of their illness according to the NYHA classification, they still presented with several unmet needs. Most of these corresponded to a lack of anticipatory care planning including a lack of discussion about concurrent PC, prognosis, advance directives, and the end of life. In addition, some patients reported receiving little attention after verbalizing symptoms that had a high impact on their quality of life and perceived a lack of support during or after acute events or at other crises points, leading them to feel unheard and alone. Finally, we identified that patients had a negative perception of the term “palliative care” and therefore feared a PC referral. Nonetheless, after a brief explanation about what concurrent and early PC involves, they were eager to know more about it and were more open to it.

### Clinical Implications

#### Systematic Screening of Unmet Needs

Participants were well-informed about the medical management of their disease because that is the routine focus of their consultations. However, possibly because there is no systematic screening of unmet needs, participants remained unaware that their unmet needs were potentially relevant to their HF care, and only upon questioning of potential specific unmet needs, they voiced how those aspects were lacking discussion. Similarly, a systematic review assessing whether end-of-life care discussions were being held with patients with HF found that the great majority of patients had not discussed their prognosis, cardiopulmonary resuscitation or other life-sustaining interventions, or plans for future care with their healthcare professionals (14). Patients can thus remain under the false impression that anything that is important to their treatment would have been covered by the cardiologist, when in reality something important to them was missing in the consultation. Therefore, a systematic screening of unmet needs carried out in each HF clinic with a tool or questionnaire developed for this purpose (15, 16) could help introduce these topics as part of the routine within HF clinics and could help identify and meet patient and family needs at a more meaningful time than is usually done at present.

There might not be enough time to assess unmet needs of patients and their caregivers within the typical 10–15 min follow-up visit with the cardiologist and there are not enough PC specialists to assess the needs of all patients in the HF clinics. However, PC specialists are not necessary for routine needs screening. One solution could be the presence of a person with PC training (a HF nurse or general practitioner) in each HF clinic who periodically examines the needs of patients and, if necessary, treats them or refers them to a specialist, as appropriate. Similar models have been implemented for the identification of needs for the delivery of PC in the ambulatory management of patients with HF (17, 18). Finally, referrals to general or specialized PC do not mean that all efforts to treat patients in a comprehensive manner are delegated to the person providing PC. All other members of the multidisciplinary team should treat patients with a palliative lens across the disease trajectory (19). Therefore, cardiologists and other HF staff could benefit from training in fundamental palliative skills for patients with HF including basic management of both physical and emotional symptoms, discussions about treatment goals, and referral to specialized PC (20, 21).

#### Anticipatory Care Planning in the Ambulatory Setting

Despite their satisfaction with the ambulatory HF clinic and its multidisciplinary approach, the lack of discussion about prognosis and future treatments led patients to have negative care experiences in other care settings, particularly during hospitalizations. In the absence of end-of-life or advance directives discussions while the patient's condition is stable, patients were not prepared for a decision making process when there was an acute cardiac decompensation. They were suddenly confronted with these types of discussions without any preparation, which often generated additional and preventable trauma. The fact that participants were dissatisfied with the way end-of-life discussions were approached is not because they are not open to such discussions. Therefore, training in anticipatory care planning for cardiologists and other members of the HF clinic team is essential to delivering high quality care (22). Likewise, following communication guides such as the about serious illness conversations might be useful for physicians to support the patient in decision making (23). Quality care must be focused not only on the setting in which the service is provided, in this case the ambulatory setting, but must also proactively prepare for the care that the patient will receive throughout the continuum of care, including other settings such as the inpatient setting.

It is common practice in hospitals that patients hospitalized for exacerbation of their illness who are not clearly terminal are asked how to proceed in case of arrest (whether to receive CPR or not). Knowing that this is standard practice in settings such as the emergency department or the ICU, it is appropriate that in outpatient management this is discussed in advance. These discussions can occur with the general practitioner as well as with the cardiologist and/or with other staff in the HF clinic. It is important to emphasize here that anticipatory care planning, the process of discussing future care strategies, is not limited to patients with a short life expectancy or with advanced HF and recommended to begin early (19). Therefore, even in HF clinics, where NYHA II patients predominate, these discussions are essential. While discussing future care strategies in the ambulatory setting, including end-of-life care, can pave the way for when advanced measures are discussed in other settings, it is equally important to make an effort in other settings to improve the way these conversations are held.

#### Normalizing the Term “Palliative Care” via It's Concurrent Use With Life-Prolonging Therapies

We identified a negative connotation of the term “palliative care”, which has been reported before in other studies among patients with cancer (24) and also with HF (25). The negative connotation of the term can provoke distress in patients with HF and their families. While we saw that explaining the concept of early and concurrent PC has a potential positive impact, if PC is only offered or discussed at the end of life, patients, families, and the community will continue to associate it with this last phase of life. Although the negative connotation of the term is a barrier to receiving it (26), health care personnel should make an effort to promote it and offering it concurrently with life-prolonging therapies, and in an early manner. This may also start at the level of the cardiology teams, as they have also been found to have misconceptions of the term which is often associated with a lack of referrals and suboptimal collaboration between PC and cardiology teams (27, 28).

### Strengths and Limitations

To our knowledge, this is the first study to elicit patient experiences exclusively from a HF clinic. We highlight two strengths of our study, which are: (1) we focused on the needs of patients from their own perspectives following a structured guide for assessing needs in patients with HF; (2) participant inclusion criteria were broad, including a representative population of the HF clinic.

Although the data was collected with patients from the same HF clinic, the results of this study can be extrapolated to similar contexts, taking into consideration the aspects of our setting and of our participant sample. Specifically, it is known that as the NYHA class increases, patients are at greater risk of frequent hospitalizations (29), depression (30), and poorer quality of life (31, 32). Therefore, it is likely that the PC needs we identified become even more relevant with increasing NYHA (33). Since the majority of participants in our study where classify as NYHA II, our study could not identify the needs of patients with more advanced HF stages and thus assess the potential role of a PC provider in HF clinics in people at advanced stages. However, the low proportion of patients classified as NYHA IV is common in ambulatory HF clinics (5, 33, 34), either because good pharmacological and non-pharmacological management keeps the functional class under control or because patients classified as NYHA IV despite optimal treatment are in other types of programs such as the transplant list program. Therefore, a lack of patients with a NYHA IV class should not hinder the transferability of our results to other ambulatory HF clinics.

The participants included in this qualitative study are a subsample of the patients included in the validation study of the German NAT: PD-HF (8). Since patients participated on a voluntary basis, patients who had negative experiences or felt that something was missing in their care may have been more inclined to participate in the study. The effect of self-selection bias can be circumvented by enhancing trustworthiness and rigor in the conduct, analysis and reporting of the study findings by following the established procedures that we have engaged with and reported at the different research phases (35). In addition, with our findings we are not suggesting that unmet PC needs are found in all HF patients, but that they can present in some of them and that careful screening by trained personnel can help with their identification.

As this is a secondary analysis of data collected from a structured interview, the main limitation of this study is the lack of depth of some of the interviews, which prevented a deeper understanding of the implications of the unmet needs of patients with HF or of their preferences for meeting those needs. However, a major advantage of the structured approach to interviewing is that all topics were addressed in all interviews uniformly, allowing us to be certain of the types of needs that were met or unmet in this patient population.

While this study focused on understanding the unmet needs of patients with heart failure from their own perspective, having the perspective of the family and caregivers would have added depth to certain themes or subthemes and helped put certain needs in context.

Today, women continue to be underrepresented in cardiology studies (36). The underrepresentation of women may affect the transferability of our results since women with HF are usually older than men with HF, have more comorbidities (and therefore more symptoms) such as kidney disease, diabetes, and hypertension (37, 38), and have more often HF with preserved ejection fraction. Although various types of drugs have been shown to improve symptoms in patients with reduced ejection fraction, little or no effectiveness has been found in patients with preserved ejection fraction (39, 40). Therefore, the latter might have different types of needs than patients with reduced ejection fraction. Additionally, as the primary caregiver is usually the partner and women outlive men, women tend to have fewer informal caregivers and require more support (41). Although in our study women were slightly more likely to accept participation than men (Supplementary Material 3), due to the low representation of female patients in the HF clinic at our institution we did not achieve adequate representation of women in our study.

## Conclusion

Although patients who are receiving multidisciplinary management in ambulatory HF clinics are generally satisfied with the care received, they remain with unmet needs. These unmet needs, such as the need for advance care planning or the need for timely and tactful end-of-life discussions, can be fulfilled by PC providers. Though patient perceptions of PC may be a challenge, including personnel trained in PC as part of the multidisciplinary team to apply tools or questionnaires to systematically assess unmet needs, could help to address these needs, thus improving both the quality of care and the quality of life of people living with HF.

## Data Availability Statement

The raw data supporting the conclusions of this article will be made available by the authors, without undue reservation.

## Ethics Statement

The studies involving human participants were reviewed and approved by BASEC-Swissethics. The patients/participants provided their written informed consent to participate in this study.

## Author Contributions

NL and JG collected the data. VG-J and SZ performed the analysis, drafted the manuscript, and responsible for the overall content of the manuscript. MM contributed to the analysis of results. MM, NL, JG, LH, and SE did a critical revision of the manuscript. All authors discussed the results and commented on the manuscript. All authors contributed to the article and approved the submitted version.

## Funding

This project was supported by Stiftung Lindenhof Bern, Teaching and Research Fund (grant numbers: 20-03-F and WRO-013). The funder had no role on the study design, data collection, analysis, or interpretation of the results.

## Conflict of Interest

The authors declare that the research was conducted in the absence of any commercial or financial relationships that could be construed as a potential conflict of interest.

## Publisher's Note

All claims expressed in this article are solely those of the authors and do not necessarily represent those of their affiliated organizations, or those of the publisher, the editors and the reviewers. Any product that may be evaluated in this article, or claim that may be made by its manufacturer, is not guaranteed or endorsed by the publisher.
