# Supplementary material for: Unmet Needs in Patients With Heart Failure: The Importance of Palliative Care in a Heart Failure Clinic
Source: Front Cardiovasc Med. 2022 May 30;9:866794. doi: 10.3389/fcvm.2022.866794 (PMC9195498; doi:10.3389/fcvm.2022.866794)

Supplementary Material

# Supplementary material 1. Original version (English) of the “Needs Assessment Tool: Progressive Disease-Heart Failure (NAT: PD-HF)”.


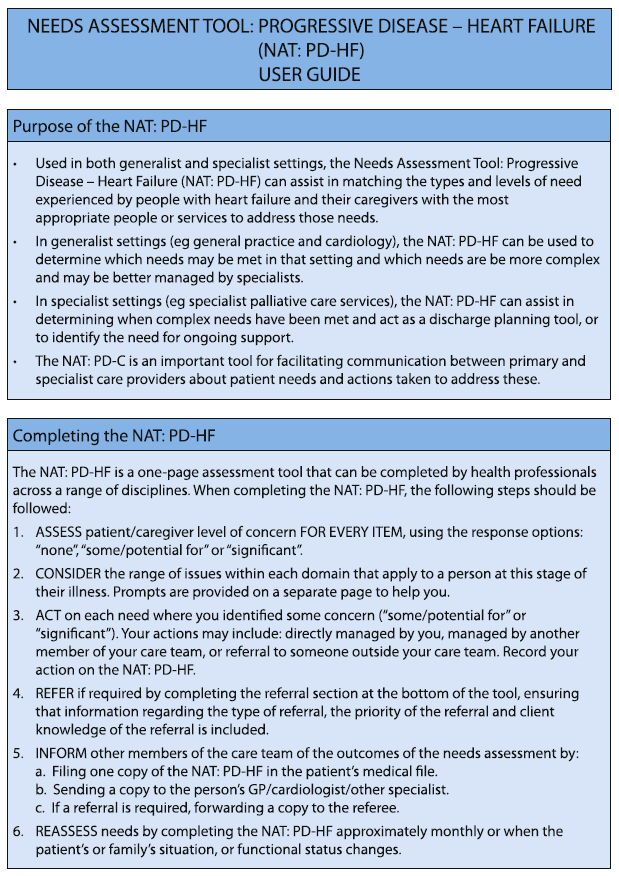


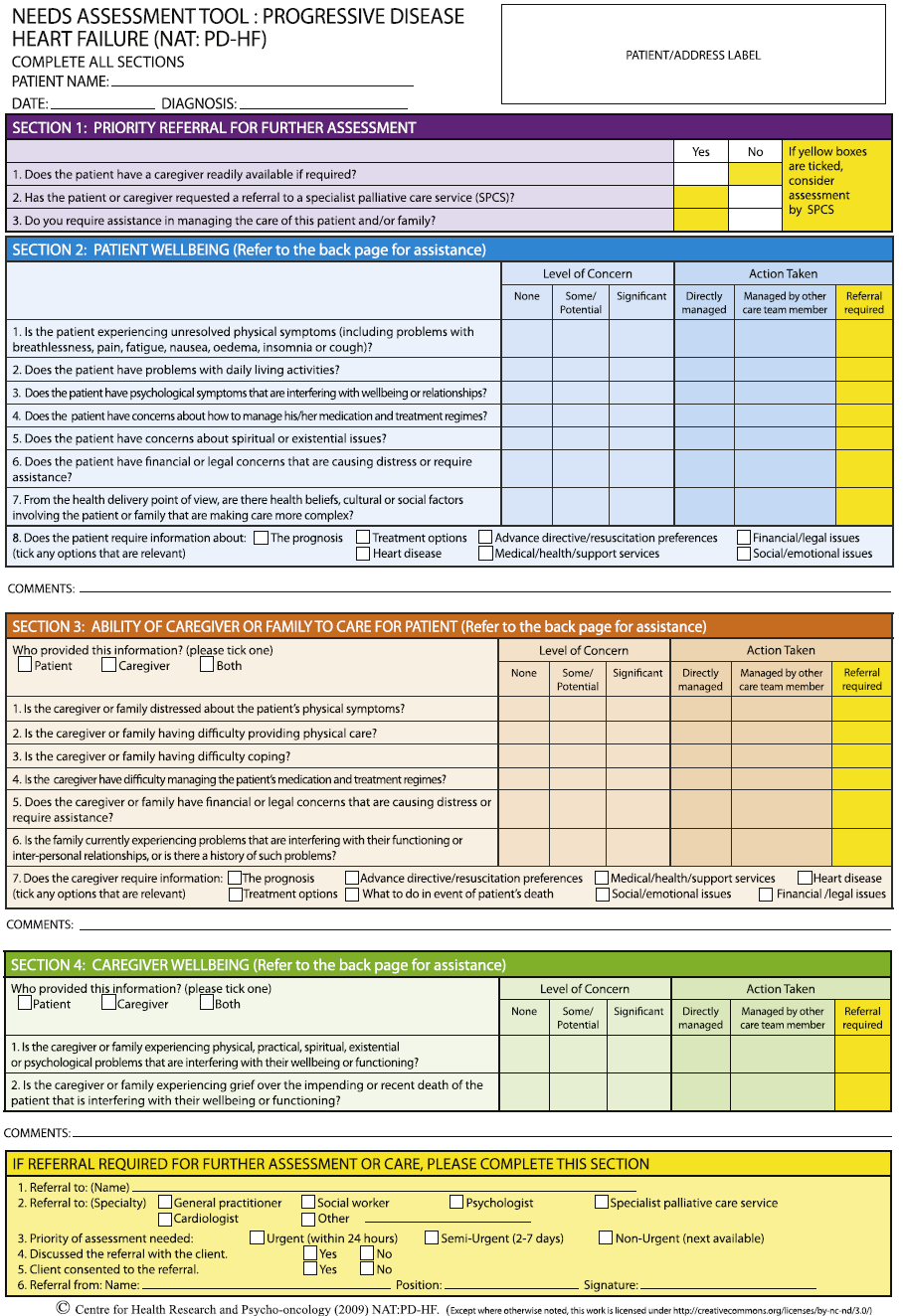


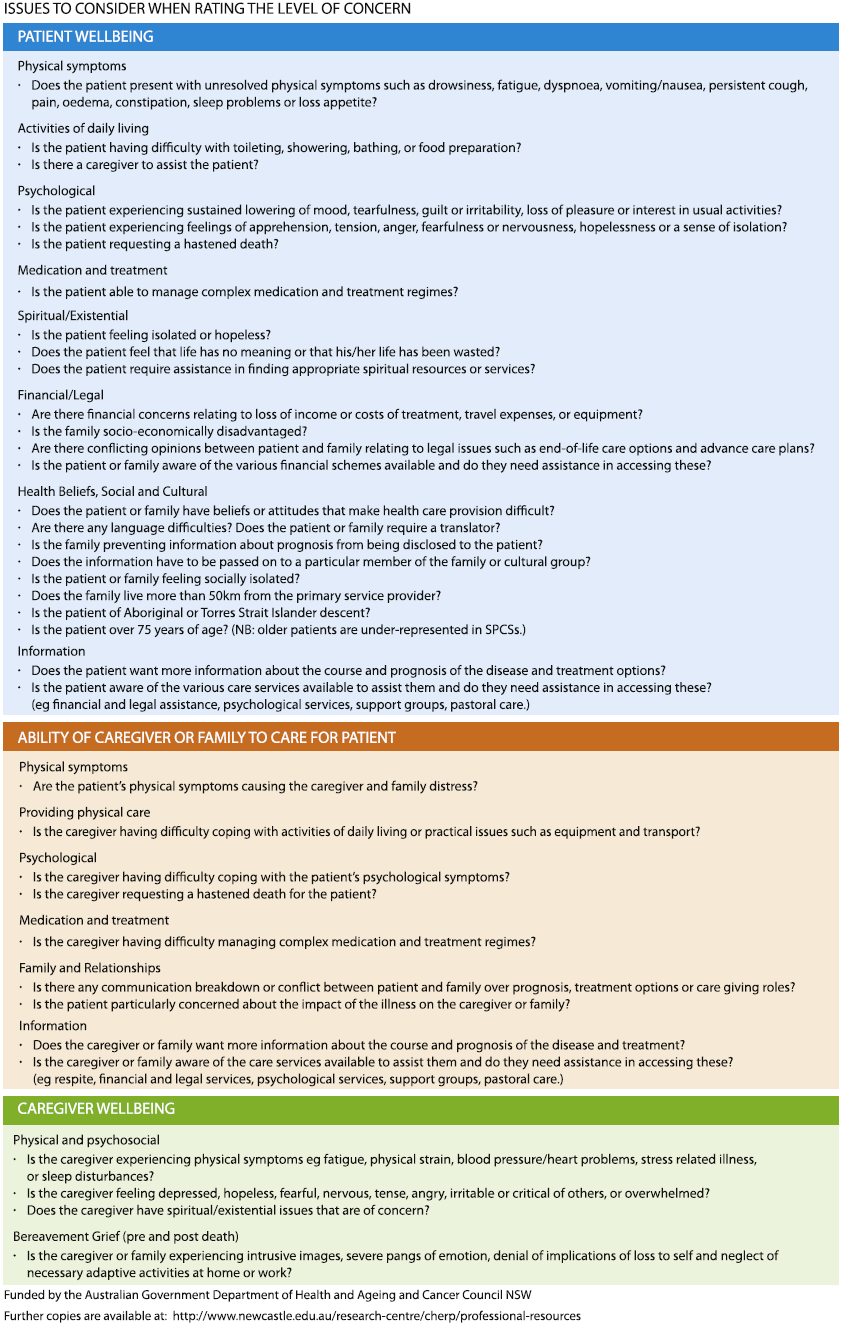


# Supplementary material 2. Characteristics of the patients included in the validation study of the German NAT: PD-HF (n=70)

| **Sociodemographic characteristics** | **n (%) or median (IQR)** |
| --- | --- |
| Women | 12 (17%) |
| Age | 62 (54-72) |
| LVEF (%) | 35 (20-50) |
| LVEF category |  |
| HFrEF | 45 (64%) |
| HFmrEF | 3 (4%) |
| HFpEF | 22 (32%) |
| NYHA functional class |  |
| I | 22 (31%) |
| II | 35 (50%) |
| III | 13 (19%) |
| ICD | 38 (54%) |
| VAD | 12 (17%) |
| Heart transplant list | 9 (13%) |
| COPD | 11 (16%) |
| CAD | 30 (43%) |
| CKD | 32 (46%) |
| IQR: interquartile rate; LVEF: left ventricular ejection fraction; ICD: implantable cardioverter defibrillator; VAD: ventricular assist device; COPD: chronic obstructive pulmonary disease; CAD: coronary artery disease, CKD: chronic kidney disease | |

# Supplementary material 3. Flowchart of the participants included in the validation study of the German NAT: PD-HF


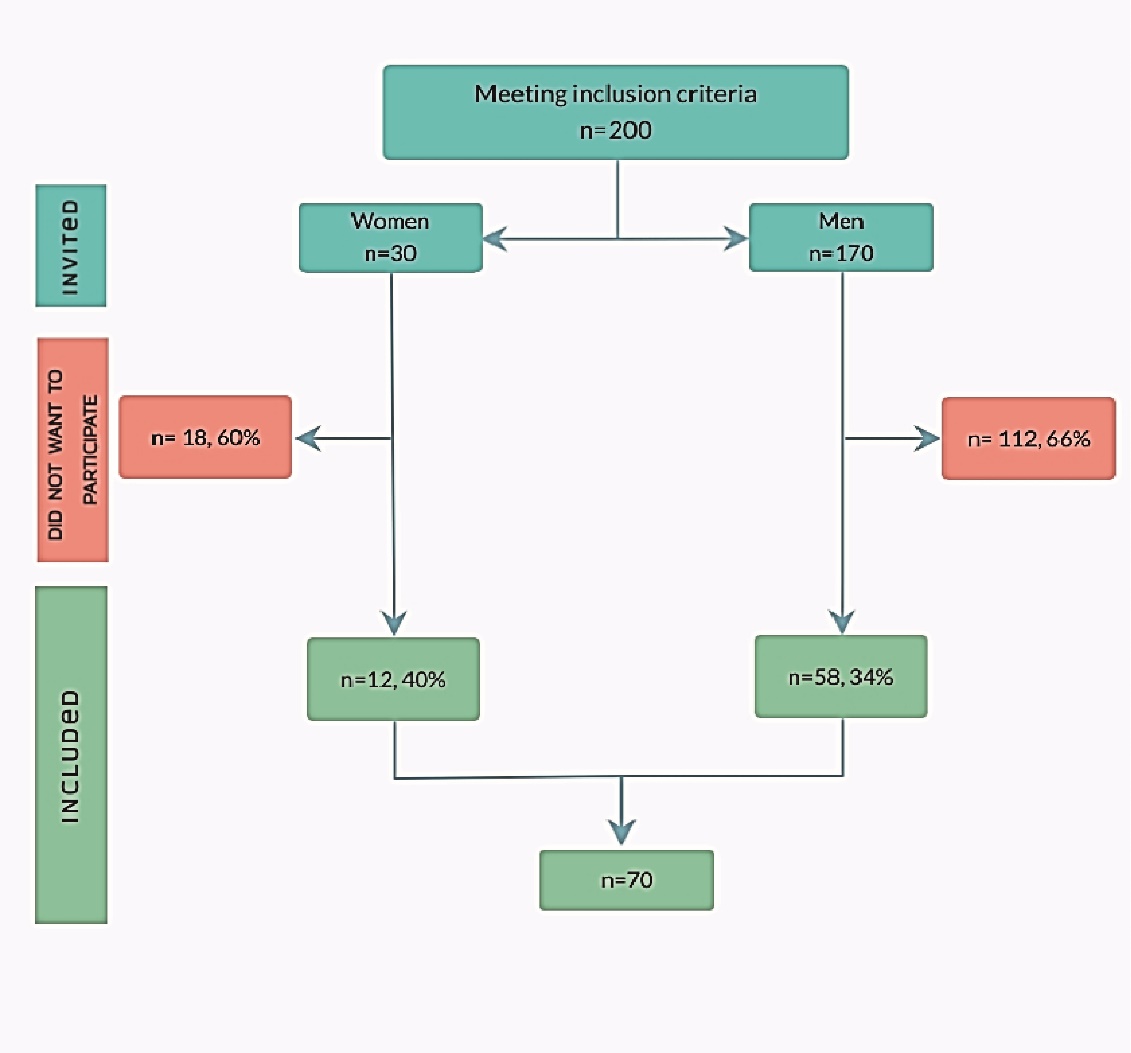

Supplement: Supplementary file 1 [file Data_Sheet_1.docx]
